# Harnessing the digital potential of the next generation of health professionals

**DOI:** 10.1186/s12960-021-00591-2

**Published:** 2021-04-14

**Authors:** Brian L. H. Wong, Mark P. Khurana, Robert D. Smith, Omnia El-Omrani, Ave Pold, Amine Lotfi, Charlotte A. O’Leary, Diah S. Saminarsih

**Affiliations:** 1 Medical Research Council (MRC) Unit for Lifelong Health and Ageing at University College London (UCL), Department of Population Science and Experimental Medicine, UCL Institute of Cardiovascular Science, University College London, 5th Floor, 1-19 Torrington Place, Fitzrovia, London, WC1E 7HB UK; 2 Global Health Workforce Network (GHWN) Youth Hub, World Health Organization, Geneva, Switzerland; 3 International Federation of Medical Students’ Associations (IFMSA), Nørre Allé 14, 2200 København N., Denmark; 4 Faculty of Health and Medical Sciences, University of Copenhagen, Copenhagen, Denmark; 5 JOHO, Youth Health Think Tank, Copenhagen, Denmark; 6 Department of Anthropology and Sociology, Graduate Institute of International and Development Studies, Geneva, Switzerland; 7 Faculty of Medicine, Ain Shams University, Ramsis Street, Abbassia, Cairo, Egypt; 8 International Association of Dental Students (IADS), Avenue Casai 51, 1216 Cointrin, Switzerland; 9 Charité-Universitätsmedizin Berlin, Charitépl. 1, 10117 Berlin, Germany; 10 Faculty of Medicine and Pharmacy of Casablanca, Hassan II University, Casablanca, Morocco; 11 The Royal Melbourne Hospital, 300 Grattan St., Parkville, VIC 3050 Australia; 12 Women in Global Health, Washington, DC USA; 13 Office of the Director-General, World Health Organization, Avenue Appia 20, 1202 Geneva, Switzerland

**Keywords:** Capacity building, Digital education, Digital ethics, Digital health, Digital literacy, Healthcare professionals, Health literacy, Public health, Youth

## Abstract

Digital technologies are rapidly being integrated into a wide range of health fields. This new domain, often termed ‘digital health’, has the potential to significantly improve healthcare outcomes and global health equity more broadly. However, its effective implementation and responsible use are contingent on building a health workforce with a sufficient level of knowledge and skills to effectively navigate the digital transformations in health. More specifically, the next generation of health professionals—namely youth—must be adequately prepared to maximise the potential of these digital transformations. In this commentary, we highlight three priority areas which should be prioritised in digital education to realise the benefits of digital health: capacity building, opportunities for youth, and an ethics-driven approach. Firstly, capacity building requires educational frameworks and curricula to not only be updated, but to also place an emphasis on interdisciplinary learning. Secondly, opportunities are important for youth to meaningfully participate in decision-making processes and gain invaluable practical experiences. Thirdly, training in digital ethics and the responsible use of data as a standard component of education will help to safeguard against potential future inequities resulting from the implementation and use of digital health technologies.

## Introduction

Digital health is becoming a pervasive component of healthcare practice, with applications in fields spanning from public health to primary healthcare and cutting-edge specialist practices [1]. Digital health, defined as “the use of digital technologies for health”, encompasses a wide-ranging umbrella which comprises the application of information and communication exchange [2]. Amidst the ongoing COVID-19 pandemic, the unprecedented potential of digital health technologies for improving access to and quality of essential health services has been proven and augmented through engaging youth in promoting their effective use [3, 4].

In May 2019, the World Health Organization (WHO) established its first digital health technical advisory group [5], building on the resolution adopted at the Seventy-first World Health Assembly in 2018 which highlighted the important role of digital health in achieving the Sustainable Development Goals [6]. Recently, the WHO also published a report titled “Youth-centred digital health interventions: a framework for planning, developing and implementing solutions with and for young people”, aimed at providing guidance for future youth-focused and youth-led digital health solutions [7]. The adoption of digital health policies also has implications on the ability of health strategies to increase access to underserved communities. Recent estimates suggest that “97% of the world’s population [lives] within reach of a mobile cellular signal” [7], yet roughly 50% of the world’s population is still offline; this highlights the potential to increase access to health services if last mile infrastructures are put in place. Conscious of the large investments required to fully realise digital health infrastructures, in the interim, younger generations can play key roles in making use of existing technology infrastructures to bridge the digital divide as they are often the most frequent and literate users of digital health technologies. This further underscores the global need to prepare for a new wave of digital health solutions. However, beyond this optimistic potential, if governance safeguards are not designed with youth ownership as a core principle, “private companies or political groups … [can] exploit” youth who have limited “power to control” their digital environments and further exacerbate the detriments of the digital divide [8].

The effective implementation and responsible use of digital health technologies is contingent on building a health workforce with the knowledge and skills to maximise its potential. In its *Draft global strategy on digital 2020–2025*, the WHO proposes to “place people at the centre of digital health through the appropriate adoption and use of digital health technologies and development of appropriate literacy” [9]. As a result of the high digital engagement and literacy of youth, they are in prime positions to understand the fundamental needs to successfully implement digital health. To realise this vision for the future, we must be well-positioned to fulfil the potential of the most digitally literate generation to date—namely, youth. Capacity building, opportunities for youth, and an ethics-driven approach are critical to realising the goal of the future health workforce being adept at navigating digital transformations in health and the improvement of health for all, all of which aligns with global goals such as universal health coverage (UHC).

## The need for capacity building and digital literacy

Firstly, capacity building and the development of digital literacy will be integral to ensuring that digital health tools are used correctly and competently in practice. Multidimensional approaches must be at the centre of capacity building initiatives; interprofessional and interdisciplinary education can also play a key role to realise this. Indeed, digital health tools will only be effective once a common knowledge base exists amongst different professions and disciplines, allowing them to integrate their respective competencies into a shared understanding which ultimately benefits patients. In this respect, successful implementation of digital health requires end-user buy-in, from healthcare professionals—to see the tangible results. To accomplish this, the enthusiasm of youth populations can play a large role in explaining the benefits of digital health interventions to the broader health workforce.

Education in digital health will guide current and future health professionals in identifying the most appropriate contexts for digital health uptake. Educational frameworks and curricula should be updated to include modules on digital health and its integration into current practice. In fact, examples of curricula updates in these fields already exist [10–12], but they must be adopted in a ubiquitous and geographically broad fashion to facilitate sustainable implementation. Such updates should be done collaboratively and with youth ownership, tailored to local contexts, and applied for healthcare workers ranging from dentists to public health professionals to community health workers. Equally important is the notion that digital health is by no means a panacea or silver bullet for all health problems.

Capacity building for digital health also requires strong governance coordination. Beyond ministries of health, other government ministries also have stakes in digital health; this ranges the ministries of information and communications technology to ministries of internal affairs, and ministries of education. Respectively, each ministry plays a key role in building foundational digital infrastructures, establishing safeguards for data security and privacy, and crafting education policy which allows for capacity building of health professionals in line with inter-ministry goals. The recent WHO Digital Implementation Investment Guide (DIIG) highlights how different ministries must work together by placing health at the centre of discussions explicitly earmarked for inter-ministry coordination towards achieving health goals [13]. In striving for multi-dimensional approaches to capacity building, an emerging concept in the literature and reaffirmed in the DIIG has been ‘enterprise architecture’, which carefully considers stakeholders’ incentive structures across digital health implementations and strives for interoperability.

Governments, ministries, and other stakeholders must also create and invest in the necessary infrastructures for educational initiatives to be launched successfully and adopted widely. Sufficient resources must be allocated specifically to the implementation of health workforce development initiatives. Partnerships between youth stakeholders—such as youth organisations—and government representatives can result in educational initiatives that are more tailored to younger audiences, improving the relevance and uptake of information. Young health professionals can also function as digital navigators who guide patients, citizens, and other healthcare professionals in developing and utilising digital tools both within and outside of clinical environments [14]. Without ambitious leadership and commitments to health budgets, promises of a highly digitally literate and competent future health workforce will fail to materialise. To accomplish this, the WHO states that the key to implementing multi-dimensional approaches to strengthen digital health capacity is to “align incentives for health workforce education and healthcare provision with public health goals and population needs” [15].

## Shaping meaningful youth policy ownership: learning through practical experiences

Secondly, youth must be afforded opportunities to participate in decision-making processes in addition to practical experiences in the field. Communication and consultative channels must be established and maintained to meaningfully amplify youth voices in the design, development, implementation, and evaluation of policies related to digital health. To avoid tokenism, policy makers must be willing to hear and act upon input from a wide range of youth voices as well as invest in accessing voices which may otherwise be excluded. On a global level, a helpful step would be to strengthen and expand the WHO’s Global Health Workforce Network Youth Hub mandate to include youth voices in deliberations regarding digital health and health workforce education. Locally, governments and health ministries should set aside resources to establish avenues for continuous collaborative efforts with youth and youth organisations including providing platforms for youth to give input on strategic and policy issues related to digital health, co-creating education and advocacy campaigns with youth organisations, and providing spaces for youth lobbying and advocacy to take place.

A common framework for locally-driven youth involvement is user-centred design; this implies youth engagement and youth ownership of digital health designs, which are enhanced by the unique digital literacy of youth [15, 16]. For example, a common success story is UNICEF's *U-Report*, a social messaging tool which surveys wide populations of youth for the purpose of collecting “data and insights [that] are shared back with communities and connected to policy makers who make decisions that affect young people” [17]. However, others have cautioned that while gathering youth opinions is helpful, they may often be left unconsidered and instead regarded as ‘incommensurable’ with existing political and/or economic interests [18]. This highlights the fact that meaningful youth engagement demands youth ownership and power, beyond just participation. Furthermore, it is also important to recognise the role which existing youth networks and activism can play. For example, the recent SARS protests in Nigeria have mobilised many low-bandwidth networks to organise youth, ensured access to health services, provided legal representation and economic support, and more [19]. Such examples of youth activism provide important lessons for governments to better understand the contextual realities and potentials of existing digital practices within countries; yet, without youth stakes in governance, these experiences are often sidelined and instead viewed as being ‘beyond the scope’ of traditional governance mechanisms.

With respect to concrete opportunities, youth must be afforded practical experiences in the health sector, through which they can translate insights into concrete action. This presents a double opportunity for policy makers to not only co-create digital health solutions that are tailored to youth, but also for youth to obtain invaluable experiences in co-creating digital health solutions, an investment that will greatly improve the capacity of the next generation of health professionals moving forward. A great example is Fondation Botnar's *OurCity* initiative, which aims to leverage youth participation as well as digital tools and transformations to improve youth health and well-being [20]. Youth Development Labs (YLabs), an interdisciplinary organisation advocating for innovation in partnership with youth, suggests emphasising safety and fun as key ingredients for a youth-driven process [21]. Safety and fun are achieved through the creation of safe spaces for open dialogue to take place and the encouragement of relaxing and engaging activities which stimulate participants. In addition, hack-a-thons, mentorship programmes, and development initiatives for young people interested in digital health can similarly bolster participation and interdisciplinary collaboration.

## An emphasis on ethics: towards meaningful and equitable digital health connectivity

Thirdly, given the rapid pace of development in the field, a focus on ethics will not only be crucial to ensuring the long-term sustainable and equitable use of digital health technologies, but also that these advances maintain and improve principles of health justice and equity. By tailoring educational programmes to local contexts, the next generation of health professionals will be better equipped to solve and prevent issues such as differences in access to digital health technologies and algorithm biases, which are examples of how digital health can exacerbate existing health inequities [22, 23]. Those youth left disconnected within the digital divide are the most vulnerable; to this end, as digital infrastructures develop experts have advocated for the use of the concept of ‘meaningful connectivity’, which analyses how digital technologies manifest in social worlds for better or worse [24]. Given the large amounts of data being generated at present—which will only increase over time—a strong focus on “governance, quality, safety, standards, privacy and data ownership” training, as highlighted by the WHO, will be crucial characteristics to successfully accomplishing ‘meaningful digital health connectivity’. This focus on digital health ethics as well as the responsible use of data in training and education will help protect against future inequities.

In addition to the normative ethical frameworks currently discussed in policy, youth populations have also effectively brought attention to the coloniality of health data. Specifically, digital identification systems in post-colonial countries have histories of use which have segregated populations to identify forced labourers and, as such, have ideologies of oppression embedded within them. If reflections on the coloniality of national data systems are not considered, digital health interventions which rely on existing national data infrastructures can dangerously exacerbate inequities via the infiltration of colonial ideologies within digital health data [25]. For digital health, movements to decolonise data may productively be thought of alongside those for decolonising global health which has a large, existing youth presence [26].

## Conclusion

As young health professionals, we are in a unique position to positively transform health through the effective implementation of digital health. While the potential of digital health technologies is enormous, it will not come to fruition without ensuring that the next generation of health professionals is capable of utilising it effectively and responsibly. This next generation are the most digitally literate to date, sometimes even referred to as ‘digital natives’, highlighting the potential of future health professionals as torchbearers for digital health. Capacity building, opportunities for youth, and an emphasis on ethics are crucial to ensuring that youth are well-equipped to fulfil this potential. It is a timely opportunity we can ill afford to miss.

## Data Availability

Not applicable.
